# *N*-(2,6-Dimethyl­phen­yl)-2,2-diphenyl­acetamide

**DOI:** 10.1107/S1600536812013451

**Published:** 2012-04-04

**Authors:** Hoong-Kun Fun, Tze Shyang Chia, Prakash S. Nayak, B. Narayana, B. K. Sarojini

**Affiliations:** a X-ray Crystallography Unit, School of Physics, Universiti Sains Malaysia, 11800 USM, Penang, Malaysia; b Department of Studies in Chemistry, Mangalore University, Mangalagangotri 574 199, India; c Department of Chemistry, P. A. College of Engineering, Nadupadavu, Montepadavu, PO, Mangalore 574 153, India

## Abstract

In the title compound, C_22_H_21_NO, the dihedral angle between the phenyl rings is 82.59 (7)°. The dimethyl­benzene ring forms dihedral angles of 52.86 (4) and 49.65 (5)° with the two phenyl rings. In the crystal, mol­ecules are linked by N—H⋯O hydrogen bonds, forming a *C*(4) chain along the *c* axis. The crystal also features C—H⋯π inter­actions.

## Related literature

For the structural similarity of *N*-substituted 2-aryl­acetamides to the lateral chain of natural benzyl­penicillin, see: Mijin & Marinkovic (2006 ▶); Mijin *et al.* (2008 ▶). For the coordination abilities of amides, see: Wu *et al.* (2008 ▶, 2010 ▶). For related structures, see: Praveen *et al.* (2011*a* ▶,*b* ▶,*c* ▶); Fun *et al.* (2011*a* ▶,*b* ▶). For reference bond lengths, see: Allen *et al.* (1987 ▶). For the stability of the temperature controller used in the data collection, see: Cosier & Glazer (1986 ▶).
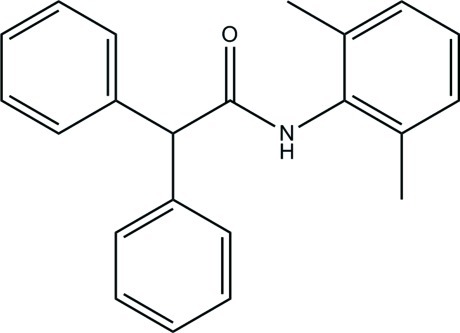


## Experimental

### 

#### Crystal data

C_22_H_21_NO
*M* *_r_* = 315.40
Monoclinic, 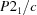

*a* = 12.0606 (10) Å
*b* = 16.6747 (13) Å
*c* = 8.9469 (7) Å
β = 108.080 (2)°
*V* = 1710.4 (2) Å^3^
*Z* = 4
Mo *K*α radiation
μ = 0.07 mm^−1^
*T* = 100 K
0.56 × 0.21 × 0.12 mm

#### Data collection

Bruker SMART APEXII CCD area-detector diffractometer
Absorption correction: multi-scan (*SADABS*; Bruker, 2009 ▶) *T*_min_ = 0.959, *T*_max_ = 0.991
19127 measured reflections
4994 independent reflections
3661 reflections with *I* > 2σ(*I*)
*R*_int_ = 0.049

#### Refinement

*R*[*F* ^2^ > 2σ(*F* ^2^)] = 0.056
*wR*(*F* ^2^) = 0.139
*S* = 1.03
4994 reflections
223 parameters
H atoms treated by a mixture of independent and constrained refinement
Δρ_max_ = 0.37 e Å^−3^
Δρ_min_ = −0.27 e Å^−3^

### 

Data collection: *APEX2* (Bruker, 2009 ▶); cell refinement: *SAINT* (Bruker, 2009 ▶); data reduction: *SAINT*; program(s) used to solve structure: *SHELXTL* (Sheldrick, 2008 ▶); program(s) used to refine structure: *SHELXTL*; molecular graphics: *SHELXTL*; software used to prepare material for publication: *SHELXTL* and *PLATON* (Spek, 2009 ▶).

## Supplementary Material

Crystal structure: contains datablock(s) global, I. DOI: 10.1107/S1600536812013451/is5102sup1.cif

Structure factors: contains datablock(s) I. DOI: 10.1107/S1600536812013451/is5102Isup2.hkl

Supplementary material file. DOI: 10.1107/S1600536812013451/is5102Isup3.cml

Additional supplementary materials:  crystallographic information; 3D view; checkCIF report

## Figures and Tables

**Table 1:** Hydrogen-bond geometry (Å, °) *Cg*1 is the centroid of the C1–C6 ring.

*D*—H⋯*A*	*D*—H	H⋯*A*	*D*⋯*A*	*D*—H⋯*A*
N1—H1*N*1⋯O1^i^	0.88 (2)	1.97 (2)	2.8207 (16)	163.2 (17)
C12—H12*A*⋯*Cg*1^ii^	0.95	2.80	3.6981 (17)	158

Symmetry codes: (i) 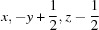; (ii) 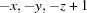.

**Fig. 1.**
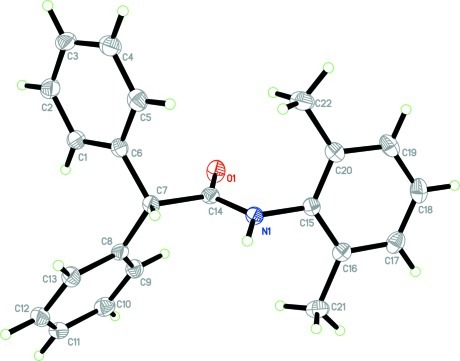
The molecular structure of the title compound with atom labels with 50% probability displacement ellipsoids.

**Fig. 2.**
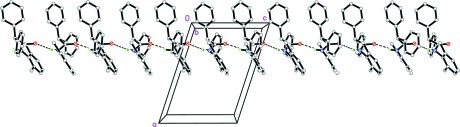
The crystal packing of the title compound. The dashed lines represent the hydrogen bonds.

## supplementary crystallographic information

### Comment

*N*-Substituted 2-arylacetamides are very interesting compounds because of their structural similarity to the lateral chain of natural benzylpenicillin (Mijin & Marinkovic, 2006; Mijin *et al.*, 2008). Amides are also used as ligands due to their excellent coordination abilities (Wu *et al.*, 2008, 2010). Crystal structures of some acetamide derivatives *viz.*, *N*-(4-chloro-1,3-benzothiazol-2-yl)-2-(3-methylphenyl)acetamide monohydrate, *N*-(3-chloro-4-fluorophenyl)-2,2-diphenylacetamide and *N*-(3-chloro-4-fluorophenyl)-2-(naphthalen-1-yl)acetamide (Praveen *et al.*, 2011*a*,*b*,*c*) have been reported. In continuation of our work on synthesis of amides (Fun *et al.*, 2011*a*,*b*), we report herein the crystal structure of the title compound (I).

The title compound (Fig. 1) consists of two benzene rings (C1–C6 & C8–C13) and one dimethylbenzene ring (C15–C22) [maximum deviation = 0.0159 (10) at atom C22]. The dihedral angle between the two phenyl rings is 82.59 (7)°. The dimethylbenzene ring forms dihedral angles of 52.86 (4) and 49.65 (5) Å with the C1–C6 and C8–C13 phenyl rings, respectively. Bond lengths (Allen *et al.*, 1987) and angles are within normal ranges and are comparable to related structures (Fun *et al.*, 2011*a*,*b*).

In the crystal (Fig. 2), molecules are linked by intermolecular N1—H1N1···O1 hydrogen bonds (Table 1), forming an infinite chain along the *c* axis. The crystal is further stabilized by C—H···π interaction (Table 1), involving *Cg*1 which is the centroid of C1–C6 ring.

### Experimental

Diphenylacetic acid (0.212 g, 1 mmol), 2,6-dimethylaniline (0.1 ml, 1 mmol) and 1-ethyl-3-(3-dimethylaminopropyl)-carbodiimide hydrochloride (1.0 g, 0.01 mol) were dissolved in dichloromethane (20 ml). The mixture was stirred in the presence of triethylamine at 273 K for about 3 h. The contents were poured into 100 ml of ice-cold aqueous hydrochloric acid with stirring which was then extracted thrice with dichloromethane. Organic layer was washed with saturated NaHCO_3_ solution and brine solution, dried and concentrated under reduced pressure to give the title compound (I). Single crystals were grown from methylene chloride and acetone (1:1) mixture by the slow evaporation method (M.P.: 469–471 K).

### Refinement

Atom H1N1 was located in a difference Fourier map and refined freely [N—H = 0.88 (2) Å]. The remaining H atoms were positioned geometrically (C—H = 0.95, 0.98 and 1.00 Å) and refined using a riding model, with *U*_iso_(H) = 1.2 or 1.5*U*_eq_(C). A rotating group model was applied to the methyl groups. Two outliers (-2 11 8) and (0 9 7) were omitted.

### Crystal data

**Table:**

C_22_H_21_NO	*F*(000) = 672
*M**_r_* = 315.40	*D*_x_ = 1.225 Mg m^−^^3^
Monoclinic, *P*2_1_/*c*	Mo *K*α radiation, λ = 0.71073 Å
Hall symbol: -P 2ybc	Cell parameters from 4188 reflections
*a* = 12.0606 (10) Å	θ = 2.4–29.9°
*b* = 16.6747 (13) Å	µ = 0.07 mm^−^^1^
*c* = 8.9469 (7) Å	*T* = 100 K
β = 108.080 (2)°	Block, colourless
*V* = 1710.4 (2) Å^3^	0.56 × 0.21 × 0.12 mm
*Z* = 4	

### Data collection

**Table:**

Bruker SMART APEXII CCD area-detector diffractometer	4994 independent reflections
Radiation source: fine-focus sealed tube	3661 reflections with *I* > 2σ(*I*)
Graphite monochromator	*R*_int_ = 0.049
φ and ω scans	θ_max_ = 30.1°, θ_min_ = 1.8°
Absorption correction: multi-scan (*SADABS*; Bruker, 2009)	*h* = −17→16
*T*_min_ = 0.959, *T*_max_ = 0.991	*k* = −23→23
19127 measured reflections	*l* = −12→12

### Refinement

**Table:**

Refinement on *F*^2^	Primary atom site location: structure-invariant direct methods
Least-squares matrix: full	Secondary atom site location: difference Fourier map
*R*[*F*^2^ > 2σ(*F*^2^)] = 0.056	Hydrogen site location: inferred from neighbouring sites
*wR*(*F*^2^) = 0.139	H atoms treated by a mixture of independent and constrained refinement
*S* = 1.03	*w* = 1/[σ^2^(*F*_o_^2^) + (0.0585*P*)^2^ + 0.6024*P*] where *P* = (*F*_o_^2^ + 2*F*_c_^2^)/3
4994 reflections	(Δ/σ)_max_ < 0.001
223 parameters	Δρ_max_ = 0.37 e Å^−^^3^
0 restraints	Δρ_min_ = −0.27 e Å^−^^3^

### Special details

**Table:**

Experimental. The crystal was placed in the cold stream of an Oxford Cryosystems Cobra open-flow nitrogen cryostat (Cosier & Glazer, 1986) operating at 100.0 (1) K.
Geometry. All e.s.d.'s (except the e.s.d. in the dihedral angle between two l.s. planes) are estimated using the full covariance matrix. The cell e.s.d.'s are taken into account individually in the estimation of e.s.d.'s in distances, angles and torsion angles; correlations between e.s.d.'s in cell parameters are only used when they are defined by crystal symmetry. An approximate (isotropic) treatment of cell e.s.d.'s is used for estimating e.s.d.'s involving l.s. planes.
Refinement. Refinement of *F*^2^ against ALL reflections. The weighted *R*-factor *wR* and goodness of fit *S* are based on *F*^2^, conventional *R*-factors *R* are based on *F*, with *F* set to zero for negative *F*^2^. The threshold expression of *F*^2^ > σ(*F*^2^) is used only for calculating *R*-factors(gt) *etc*. and is not relevant to the choice of reflections for refinement. *R*-factors based on *F*^2^ are statistically about twice as large as those based on *F*, and *R*- factors based on ALL data will be even larger.

### Fractional atomic coordinates and isotropic or equivalent isotropic displacement parameters (Å^2^)

**Table:**

	*x*	*y*	*z*	*U*_iso_*/*U*_eq_	
O1	0.20052 (9)	0.19921 (6)	1.02094 (12)	0.0242 (2)	
N1	0.27147 (10)	0.26010 (7)	0.84282 (14)	0.0170 (2)	
C1	0.09237 (12)	0.00933 (8)	0.86256 (17)	0.0219 (3)	
H1A	0.0137	0.0271	0.8351	0.026*	
C2	0.12231 (14)	−0.06573 (9)	0.93123 (19)	0.0266 (3)	
H2A	0.0638	−0.0992	0.9488	0.032*	
C3	0.23640 (14)	−0.09192 (9)	0.97397 (19)	0.0289 (4)	
H3A	0.2566	−0.1431	1.0214	0.035*	
C4	0.32122 (14)	−0.04310 (9)	0.9472 (2)	0.0293 (4)	
H4A	0.4000	−0.0607	0.9768	0.035*	
C5	0.29162 (13)	0.03156 (9)	0.87732 (18)	0.0238 (3)	
H5A	0.3503	0.0645	0.8589	0.029*	
C6	0.17645 (12)	0.05855 (8)	0.83388 (17)	0.0185 (3)	
C7	0.14945 (11)	0.14193 (8)	0.76064 (16)	0.0174 (3)	
H7A	0.1869	0.1460	0.6757	0.021*	
C8	0.01942 (12)	0.15663 (8)	0.68561 (17)	0.0183 (3)	
C9	−0.04747 (13)	0.20118 (8)	0.75703 (18)	0.0228 (3)	
H9A	−0.0115	0.2265	0.8553	0.027*	
C10	−0.16715 (13)	0.20883 (9)	0.6849 (2)	0.0269 (3)	
H10A	−0.2122	0.2397	0.7340	0.032*	
C11	−0.22128 (13)	0.17175 (9)	0.54190 (19)	0.0257 (3)	
H11A	−0.3031	0.1767	0.4939	0.031*	
C12	−0.15501 (13)	0.12737 (9)	0.46949 (19)	0.0254 (3)	
H12A	−0.1913	0.1020	0.3714	0.031*	
C13	−0.03575 (13)	0.12027 (8)	0.54073 (17)	0.0222 (3)	
H13A	0.0093	0.0902	0.4903	0.027*	
C14	0.20834 (12)	0.20360 (8)	0.88693 (17)	0.0176 (3)	
C15	0.33925 (11)	0.31871 (8)	0.95000 (16)	0.0174 (3)	
C16	0.30565 (12)	0.39937 (8)	0.93049 (17)	0.0195 (3)	
C17	0.37580 (13)	0.45554 (9)	1.03253 (18)	0.0245 (3)	
H17A	0.3545	0.5106	1.0217	0.029*	
C18	0.47589 (13)	0.43236 (9)	1.14927 (19)	0.0271 (3)	
H18A	0.5230	0.4715	1.2171	0.033*	
C19	0.50738 (12)	0.35244 (10)	1.16725 (18)	0.0251 (3)	
H19A	0.5758	0.3370	1.2484	0.030*	
C20	0.44015 (12)	0.29401 (8)	1.06793 (18)	0.0214 (3)	
C21	0.19801 (14)	0.42557 (9)	0.80384 (19)	0.0269 (3)	
H21A	0.1777	0.4803	0.8254	0.040*	
H21B	0.1335	0.3894	0.8013	0.040*	
H21C	0.2125	0.4242	0.7020	0.040*	
C22	0.47809 (14)	0.20760 (9)	1.0863 (2)	0.0292 (4)	
H22A	0.4740	0.1854	0.9833	0.044*	
H22B	0.4265	0.1770	1.1309	0.044*	
H22C	0.5584	0.2041	1.1568	0.044*	
H1N1	0.2599 (16)	0.2654 (11)	0.742 (2)	0.030 (5)*	

### Atomic displacement parameters (Å^2^)

**Table:**

	*U*^11^	*U*^22^	*U*^33^	*U*^12^	*U*^13^	*U*^23^
O1	0.0360 (6)	0.0259 (5)	0.0127 (5)	−0.0079 (4)	0.0105 (4)	−0.0027 (4)
N1	0.0219 (5)	0.0189 (5)	0.0098 (6)	−0.0018 (4)	0.0041 (5)	−0.0006 (5)
C1	0.0240 (6)	0.0216 (6)	0.0193 (7)	0.0000 (5)	0.0055 (6)	−0.0009 (6)
C2	0.0346 (8)	0.0226 (7)	0.0204 (8)	−0.0028 (6)	0.0055 (6)	0.0023 (6)
C3	0.0405 (8)	0.0198 (6)	0.0192 (8)	0.0055 (6)	−0.0010 (7)	−0.0013 (6)
C4	0.0288 (7)	0.0279 (7)	0.0253 (8)	0.0080 (6)	0.0001 (6)	−0.0082 (7)
C5	0.0247 (6)	0.0254 (7)	0.0212 (8)	0.0006 (5)	0.0071 (6)	−0.0063 (6)
C6	0.0236 (6)	0.0192 (6)	0.0118 (6)	0.0001 (5)	0.0041 (5)	−0.0039 (5)
C7	0.0223 (6)	0.0192 (6)	0.0110 (6)	−0.0020 (5)	0.0058 (5)	−0.0022 (5)
C8	0.0247 (6)	0.0163 (6)	0.0135 (6)	−0.0011 (5)	0.0052 (5)	0.0022 (5)
C9	0.0289 (7)	0.0199 (6)	0.0188 (7)	−0.0003 (5)	0.0060 (6)	−0.0027 (6)
C10	0.0294 (7)	0.0238 (7)	0.0280 (9)	0.0045 (6)	0.0097 (7)	0.0004 (7)
C11	0.0258 (7)	0.0231 (7)	0.0244 (8)	0.0004 (5)	0.0021 (6)	0.0055 (6)
C12	0.0302 (7)	0.0264 (7)	0.0166 (7)	−0.0049 (6)	0.0028 (6)	0.0019 (6)
C13	0.0294 (7)	0.0220 (6)	0.0157 (7)	−0.0016 (5)	0.0077 (6)	−0.0025 (6)
C14	0.0209 (6)	0.0185 (6)	0.0135 (6)	0.0004 (5)	0.0054 (5)	0.0011 (5)
C15	0.0185 (6)	0.0205 (6)	0.0141 (6)	−0.0020 (5)	0.0064 (5)	−0.0001 (5)
C16	0.0235 (6)	0.0207 (6)	0.0168 (7)	0.0000 (5)	0.0097 (6)	0.0003 (6)
C17	0.0330 (7)	0.0199 (6)	0.0231 (8)	−0.0039 (6)	0.0124 (6)	−0.0027 (6)
C18	0.0296 (7)	0.0298 (7)	0.0234 (8)	−0.0110 (6)	0.0102 (6)	−0.0065 (7)
C19	0.0199 (6)	0.0362 (8)	0.0178 (7)	−0.0044 (6)	0.0041 (6)	−0.0032 (7)
C20	0.0200 (6)	0.0258 (7)	0.0187 (7)	−0.0001 (5)	0.0068 (6)	−0.0009 (6)
C21	0.0339 (8)	0.0238 (7)	0.0209 (8)	0.0071 (6)	0.0054 (7)	0.0019 (6)
C22	0.0275 (7)	0.0298 (7)	0.0255 (8)	0.0080 (6)	0.0012 (6)	−0.0006 (7)

### Geometric parameters (Å, º)

**Table:**

O1—C14	1.2337 (17)	C10—H10A	0.9500
N1—C14	1.3452 (18)	C11—C12	1.388 (2)
N1—C15	1.4340 (17)	C11—H11A	0.9500
N1—H1N1	0.88 (2)	C12—C13	1.386 (2)
C1—C6	1.389 (2)	C12—H12A	0.9500
C1—C2	1.392 (2)	C13—H13A	0.9500
C1—H1A	0.9500	C15—C16	1.3998 (19)
C2—C3	1.380 (2)	C15—C20	1.4024 (19)
C2—H2A	0.9500	C16—C17	1.395 (2)
C3—C4	1.385 (2)	C16—C21	1.499 (2)
C3—H3A	0.9500	C17—C18	1.384 (2)
C4—C5	1.389 (2)	C17—H17A	0.9500
C4—H4A	0.9500	C18—C19	1.381 (2)
C5—C6	1.3957 (19)	C18—H18A	0.9500
C5—H5A	0.9500	C19—C20	1.396 (2)
C6—C7	1.5285 (19)	C19—H19A	0.9500
C7—C8	1.5221 (18)	C20—C22	1.505 (2)
C7—C14	1.5298 (18)	C21—H21A	0.9800
C7—H7A	1.0000	C21—H21B	0.9800
C8—C9	1.389 (2)	C21—H21C	0.9800
C8—C13	1.398 (2)	C22—H22A	0.9800
C9—C10	1.392 (2)	C22—H22B	0.9800
C9—H9A	0.9500	C22—H22C	0.9800
C10—C11	1.388 (2)		
			
C14—N1—C15	122.57 (12)	C13—C12—C11	119.78 (14)
C14—N1—H1N1	116.8 (12)	C13—C12—H12A	120.1
C15—N1—H1N1	119.8 (12)	C11—C12—H12A	120.1
C6—C1—C2	120.58 (14)	C12—C13—C8	121.11 (14)
C6—C1—H1A	119.7	C12—C13—H13A	119.4
C2—C1—H1A	119.7	C8—C13—H13A	119.4
C3—C2—C1	120.45 (15)	O1—C14—N1	123.20 (13)
C3—C2—H2A	119.8	O1—C14—C7	121.33 (12)
C1—C2—H2A	119.8	N1—C14—C7	115.43 (12)
C2—C3—C4	119.52 (14)	C16—C15—C20	121.74 (13)
C2—C3—H3A	120.2	C16—C15—N1	119.24 (12)
C4—C3—H3A	120.2	C20—C15—N1	118.97 (12)
C3—C4—C5	120.27 (14)	C17—C16—C15	118.04 (13)
C3—C4—H4A	119.9	C17—C16—C21	120.39 (13)
C5—C4—H4A	119.9	C15—C16—C21	121.57 (13)
C4—C5—C6	120.58 (14)	C18—C17—C16	121.07 (14)
C4—C5—H5A	119.7	C18—C17—H17A	119.5
C6—C5—H5A	119.7	C16—C17—H17A	119.5
C1—C6—C5	118.59 (13)	C19—C18—C17	120.08 (14)
C1—C6—C7	123.10 (12)	C19—C18—H18A	120.0
C5—C6—C7	118.29 (13)	C17—C18—H18A	120.0
C8—C7—C6	112.88 (11)	C18—C19—C20	120.98 (14)
C8—C7—C14	113.34 (11)	C18—C19—H19A	119.5
C6—C7—C14	107.85 (11)	C20—C19—H19A	119.5
C8—C7—H7A	107.5	C19—C20—C15	118.09 (13)
C6—C7—H7A	107.5	C19—C20—C22	120.10 (13)
C14—C7—H7A	107.5	C15—C20—C22	121.79 (13)
C9—C8—C13	118.75 (13)	C16—C21—H21A	109.5
C9—C8—C7	123.30 (13)	C16—C21—H21B	109.5
C13—C8—C7	117.88 (13)	H21A—C21—H21B	109.5
C8—C9—C10	120.14 (14)	C16—C21—H21C	109.5
C8—C9—H9A	119.9	H21A—C21—H21C	109.5
C10—C9—H9A	119.9	H21B—C21—H21C	109.5
C11—C10—C9	120.66 (15)	C20—C22—H22A	109.5
C11—C10—H10A	119.7	C20—C22—H22B	109.5
C9—C10—H10A	119.7	H22A—C22—H22B	109.5
C10—C11—C12	119.55 (14)	C20—C22—H22C	109.5
C10—C11—H11A	120.2	H22A—C22—H22C	109.5
C12—C11—H11A	120.2	H22B—C22—H22C	109.5
			
C6—C1—C2—C3	0.9 (2)	C7—C8—C13—C12	176.47 (13)
C1—C2—C3—C4	−0.4 (2)	C15—N1—C14—O1	2.5 (2)
C2—C3—C4—C5	−0.3 (2)	C15—N1—C14—C7	−175.23 (11)
C3—C4—C5—C6	0.3 (2)	C8—C7—C14—O1	79.21 (16)
C2—C1—C6—C5	−0.9 (2)	C6—C7—C14—O1	−46.51 (17)
C2—C1—C6—C7	−179.26 (14)	C8—C7—C14—N1	−103.05 (14)
C4—C5—C6—C1	0.2 (2)	C6—C7—C14—N1	131.23 (12)
C4—C5—C6—C7	178.71 (13)	C14—N1—C15—C16	−112.23 (15)
C1—C6—C7—C8	−14.54 (19)	C14—N1—C15—C20	70.24 (18)
C5—C6—C7—C8	167.05 (13)	C20—C15—C16—C17	0.0 (2)
C1—C6—C7—C14	111.45 (15)	N1—C15—C16—C17	−177.47 (13)
C5—C6—C7—C14	−66.97 (16)	C20—C15—C16—C21	179.73 (14)
C6—C7—C8—C9	101.42 (15)	N1—C15—C16—C21	2.3 (2)
C14—C7—C8—C9	−21.56 (19)	C15—C16—C17—C18	0.2 (2)
C6—C7—C8—C13	−75.63 (16)	C21—C16—C17—C18	−179.52 (14)
C14—C7—C8—C13	161.39 (12)	C16—C17—C18—C19	−0.5 (2)
C13—C8—C9—C10	0.2 (2)	C17—C18—C19—C20	0.6 (2)
C7—C8—C9—C10	−176.82 (13)	C18—C19—C20—C15	−0.4 (2)
C8—C9—C10—C11	0.5 (2)	C18—C19—C20—C22	178.07 (15)
C9—C10—C11—C12	−0.7 (2)	C16—C15—C20—C19	0.1 (2)
C10—C11—C12—C13	0.2 (2)	N1—C15—C20—C19	177.57 (13)
C11—C12—C13—C8	0.5 (2)	C16—C15—C20—C22	−178.36 (14)
C9—C8—C13—C12	−0.7 (2)	N1—C15—C20—C22	−0.9 (2)

### Hydrogen-bond geometry (Å, º)

*Cg*1 is the centroid of the C1–C6 ring.

**Table:**

*D*—H···*A*	*D*—H	H···*A*	*D*···*A*	*D*—H···*A*
N1—H1*N*1···O1^i^	0.88 (2)	1.97 (2)	2.8207 (16)	163.2 (17)
C12—H12*A*···*Cg*1^ii^	0.95	2.80	3.6981 (17)	158

Symmetry codes: (i) *x*, −*y*+1/2, *z*−1/2; (ii) −*x*, −*y*, −*z*+1.

## References

[bb1] Allen, F. H., Kennard, O., Watson, D. G., Brammer, L., Orpen, A. G. & Taylor, R. (1987). *J. Chem. Soc. Perkin Trans. 2*, pp. S1–19.

[bb2] Bruker (2009). *SADABS*, *APEX2* and *SAINT* Bruker AXS Inc., Madison, Wisconsin, USA.

[bb3] Cosier, J. & Glazer, A. M. (1986). *J. Appl. Cryst.* **19**, 105–107.

[bb4] Fun, H.-K., Quah, C. K., Narayana, B., Nayak, P. S. & Sarojini, B. K. (2011*a*). *Acta Cryst.* E**67**, o2926–o2927. 10.1107/S1600536811041110 PMC3247340 22219958

[bb5] Fun, H.-K., Quah, C. K., Narayana, B., Nayak, P. S. & Sarojini, B. K. (2011*b*). *Acta Cryst.* E**67**, o2941–o2942. 10.1107/S1600536811041468 PMC3247353 22219971

[bb6] Mijin, D. & Marinkovic, A. (2006). *Synth. Commun.* **36**, 193–198.

[bb7] Mijin, D. Z., Prascevic, M. & Petrovic, S. D. (2008). *J. Serb. Chem. Soc.* **73**, 945–950.

[bb8] Praveen, A. S., Jasinski, J. P., Golen, J. A., Narayana, B. & Yathirajan, H. S. (2011*a*). *Acta Cryst.* E**67**, o1826. 10.1107/S1600536811024597 PMC3151961 21837194

[bb9] Praveen, A. S., Jasinski, J. P., Golen, J. A., Yathirajan, H. S. & Narayana, B. (2011*b*). *Acta Cryst.* E**67**, o2602–o2603. 10.1107/S1600536811035872 PMC3201351 22064942

[bb10] Praveen, A. S., Jasinski, J. P., Golen, J. A., Narayana, B. & Yathirajan, H. S. (2011*c*). *Acta Cryst.* E**67**, o2604. 10.1107/S1600536811036075 PMC3201262 22058752

[bb11] Sheldrick, G. M. (2008). *Acta Cryst.* A**64**, 112–122. 10.1107/S0108767307043930 18156677

[bb12] Spek, A. L. (2009). *Acta Cryst.* D**65**, 148–155. 10.1107/S090744490804362X PMC2631630 19171970

[bb13] Wu, W.-N., Cheng, F.-X., Yan, L. & Tang, N. (2008). *J. Coord. Chem.* **61**, 2207–2215.

[bb14] Wu, W.-N., Wang, Y., Zhang, A.-Y., Zhao, R.-Q. & Wang, Q.-F. (2010). *Acta Cryst.* E**66**, m288. 10.1107/S160053681000471X PMC2983540 21580233

